# Microwell bag culture for large-scale production of homogeneous islet-like clusters

**DOI:** 10.1038/s41598-022-09124-w

**Published:** 2022-03-25

**Authors:** Ryo Suenaga, Shuhei Konagaya, Junji Yamaura, Ryo Ito, Satoshi Tanaka, Yoichi Ishizaki, Taro Toyoda

**Affiliations:** 1 Corporate Research & Development, Toyo Seikan Group Holdings, Ltd., Yokohama, Japan; 2 iPSC-derived Pancreatic Islet Cells (iPIC) Therapy Department, Orizuru Therapeutics, Inc., Fujisawa, Kanagawa Japan; 3 Cell Therapies, Pharmaceutical Science, Takeda Pharmaceutical Company Limited, Fujisawa, Kanagawa Japan; 4 Department of Cell Growth and Differentiation, Center for iPS Cell Research and Application, Kyoto University, Kyoto, Japan; 5 Takeda-CiRA Joint Program for iPS Cell Applications (T-CiRA), Fujisawa, Kanagawa Japan

**Keywords:** Biomaterials, Regenerative medicine, Stem-cell biotechnology

## Abstract

Pluripotent stem-cell derived cells can be used for type I diabetes treatment, but we require at least 10^5^–10^6^ islet-like clusters per patient. Although thousands of uniform cell clusters can be produced using a conventional microwell plate, numerous obstacles need to be overcome for its clinical use. In this study, we aimed to develop a novel bag culture method for the production of uniform cell clusters on a large scale (10^5^–10^6^ clusters). We prepared small-scale culture bags (< 10^5^ clusters) with microwells at the bottom and optimized the conditions for producing uniform-sized clusters in the bag using undifferentiated induced pluripotent stem cells (iPSCs). Subsequently, we verified the suitability of the bag culture method using iPSC-derived pancreatic islet cells (iPICs) and successfully demonstrate the production of 6.5 × 10^5^ uniform iPIC clusters using a large-scale bag. In addition, we simplified the pre- and post-process of the culture—a degassing process before cell seeding and a cluster harvesting process. In conclusion, compared with conventional methods, the cluster production method using bags exhibits improved scalability, sterility, and operability for both clinical and research use.

## Introduction

Islet transplantation is a promising method for the treatment of type I diabetes, which is caused by β-cell destruction followed by insulin insufficiency. Generally, 10^5^–10^6^ islets (cell clusters of endocrine cells) are used to treat one patient; however, the scarcity of high-quality donor cells is a limiting factor for the widespread application of islet transplantation^1,2^. Recently, pluripotent stem cells (PSCs), such as embryonic stem cells and induced pluripotent stem cells (iPSCs), have become promising alternative cell sources for transplantation therapies, including islet transplantation^3–5^.

Isolated islets from donors are heterogeneous in size; small islets are superior to larger ones in terms of insulin secretion and cell survival, in vitro and in vivo^6,7^. Recent studies have demonstrated that isolated islets could be dissociated into single cells and then reaggregated using a microwell plate to make uniform-sized islets, called pseudo-islets^8,9^. These size-controlled islets improve glucose-stimulated insulin secretion and hypoxia tolerance in vitro, and their efficacy in reversing hyperglycemia is better than that of native islets^8^. In PSC-derived islets, the size of the cell cluster affects the efficacy, similar to that in native islets^10^. In addition, recent reports have shown that reaggregation contributes to the depletion of non-endocrine cells, which are undesired cells accompanying the differentiation from PSCs^11^, and promotes the functional maturity of PSC-derived β cells^12,13^. These results suggest that reaggregation is beneficial for PSC-derived islet cells.

Microwell plates have been developed to produce many uniform-sized clusters^14–16^. However, the number of clusters that can be produced at one time is generally 10^3^–10^4^, which is insufficient as a clinical dose for islet transplantation. In addition, proficiency in cell culturing techniques is required for handling microwell plates, with cell seeding and harvesting performed in an open system, which carries a high risk of contamination; thus, the use of microwell plates is not preferred for clinical application. Specifically, the aggregation step forms a part of the final preparation for transplantation, requiring a high level of sterility. Although many studies have been conducted on reconstructed islets, most are small-scale studies; there are no reports on the production of homogenous 10^5^–10^6^ islets—a clinically sufficient number of pseudo-islets—in a closed system.

Culture bags can be used for cell culture in a closed system and enable culture scale up; therefore, they have been widely used in clinical cell preparations, such as in the expansion of lymphocytes and mesenchymal stromal cells^16,17^. In this study, we developed a culture bag for large-scale production of cell clusters in a closed system. We aimed to establish a culture method that meets the requirements of clinical use by improving sterility, reproducibility, and operability, and produces a large number of uniform-sized clusters. Using iPSC-derived pancreatic islet cells (iPICs), we verified the feasibility of the microwell bag culture to produce 6.5 × 10^5^ cell clusters in a closed system.

## Results

### Optimization of microwell bag culture

**Figure 1:**
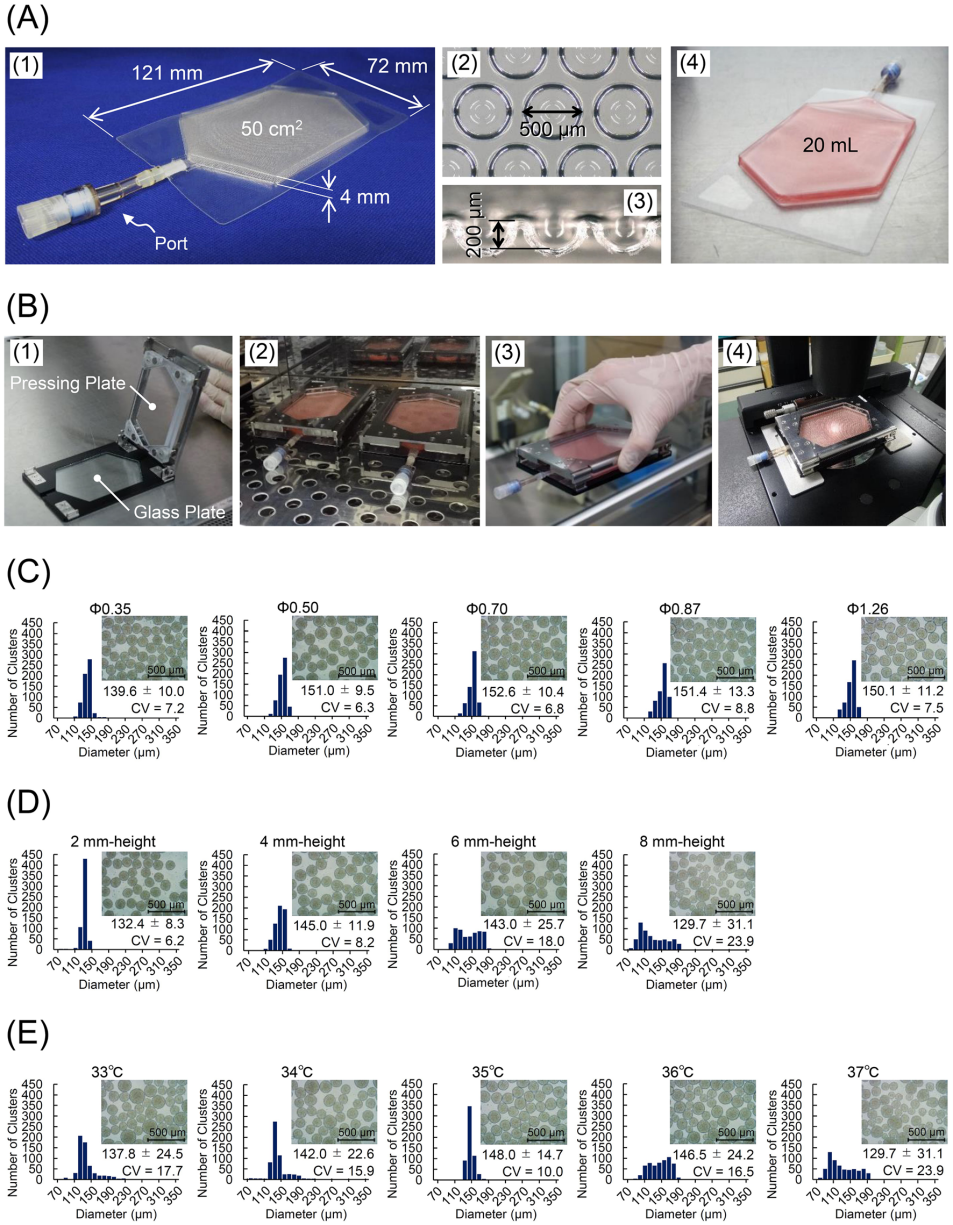
Optimization of microwell bag culture. (**A**) The design of a small-scale microwell bag. (A-1) The appearance of the small-scale microwell bag. The bag was made of a gas-permeable film and connected with a port. (A-2) Overhead view of microwells with the top film removed. (A-3) A cross-section image of microwells. (A-4) The appearance of the microwell bag containing 20 mL culture medium. (**B**) The design of a jig for a small-scale microwell bag. (B-1) The appearance of the jig with an open cover. A glass plate is inserted in the base plate, and a transparent pressing plate is attached to the cover. (B-2) Cells were cultured with the bag attached to the jig in an incubator. Displacement of the jig–bag assembly (B-3) and cell observation under a microscope (B-4) were performed in a manner similar to that for general cell culture vessels. (**C**–**E**) Distribution of cluster size after 2 day culture of human induced pluripotent stem cells (hiPSCs) in a small-scale bag. The size of the clusters was analyzed from their phase-contrast images. In total, 600 clusters were used for the measurement (data represent mean ± standard deviation), and coefficients of variation (CVs) are indicated in the histogram. The inserted photographs are representative phase-contrast images of the clusters harvested from the bags. (**C**) hiPSCs were cultured in the bags with φ 0.35 mm, φ 0.50 mm, φ 0.87 mm, and φ 1.26 mm microwells. (**D**) hiPSCs were cultured in the bag with φ 0.50 mm microwells, and heights of 2, 4, 6, and 8 mm. (**E**) hiPSCs were seeded into an 8 mm-height bag with φ 0.50 mm microwells, following suspension in the medium at 33, 34, 35, 36, or 37 °C; cells in the bag were mixed on a hot plate set to the same temperature. The result at 37 °C corresponds to 8 mm height in (D). Scale bars = 500 μm.

We designed a novel cell culture container—the microwell bag—to produce a large number of uniform-sized clusters in a closed system, and prototyped small-scale bags to optimize the design (Fig. 1A). The bag consisted of two gas-permeable films and a port: the bottom film formed microwells and the top film formed vertical walls corresponding to the height of the medium. We also designed a jig to improve the operativity of the microwell bag (Fig. 1B). The pressing plate of the jig pressed the top film from above to restrain the movement of culture solution, reducing the movement of cell clusters to other wells (Supplementary Fig. S1). In addition, the jig maintained the medium at a constant height throughout the culture area, reducing variations in the number of cells settled in each microwell after seeding (Supplementary Fig. S1). When the medium was injected into the microwell bag through its port, most of the microwells were not filled with the medium owing to trapped microbubbles, which may interfere with uniform cell seeding. However, the microbubbles could be released by applying the pressing plate of the jig through the gas-permeable film overnight (Supplementary Fig. S2 and Supplementary Video S1).

To examine a relationship between the diameter of the microwells and uniformity of the clusters, we prepared microwell bags with different numbers of wells (Supplementary Fig. S3). An adjusted number of undifferentiated human iPSCs (hiPSCs) was seeded in each bag so that the calculated number of cells per well was similar; the variability in the cluster diameter was measured after 2 days of culturing. As shown in Fig. 1C and Supplementary Fig. S3, similar size clusters were formed in each bag. The coefficient of variation (CV) for the cluster size was 6.3–8.8, indicating that the diameter of the microwell did not affect the uniformity of the clusters. Next, to identify the height of the medium in which uniform-sized clusters could be obtained, we made bags with different heights (well diameter: 500 µm) and corresponding jigs (Supplementary Fig. S4). By increasing the height, the cluster size became more heterogeneous, and the CV of the cluster size was over 15 in 6 and 8 mm high bags (Fig. 1D). As the cluster size had a tendency to be larger in the center of a bag than in the periphery (Supplementary Fig. S4), the heterogeneous size could be attributed to the rapid convection of the medium while the seeded cells settled. Supporting this hypothesis, we also found that an optimal medium temperature for cell seeding minimized the difference in cluster size between the center and the periphery of the bag (Fig. 1E and Supplementary Fig. S5). Notably, even in a bag with a height of 8 mm, uniform clusters could be produced by adjusting the temperature.

### Efficient gas permeability in proximity to cell clusters

**Figure 2:**
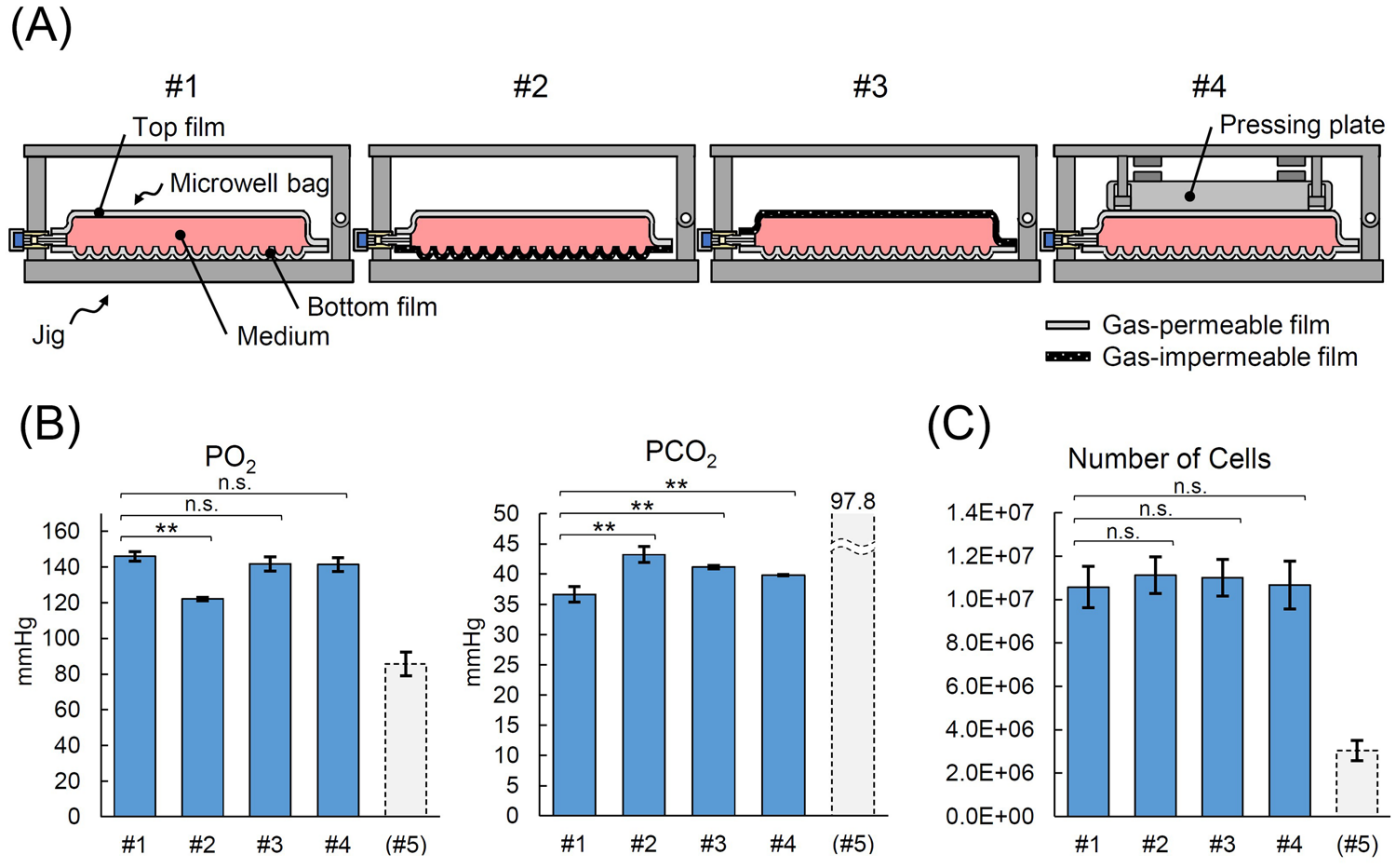
Effective gas exchange with gas-permeable films in closed culture. (**A**) A schematic illustration of the study. The top or bottom gas-permeable film of a microwell bag was replaced with a gas-impermeable film. #1: control gas-permeable bag, #2: bottom film was replaced with gas-impermeable film, #3: top film was replaced with gas-impermeable film, and #4: the top of a bag was covered with a plastic plate, which may inhibit gas exchange, to keep the medium height constant. The human induced pluripotent stem cells (hiPSCs) were cultured for 2 days using φ 0.50 mm bags. (**B**) Oxygen- and carbon dioxide partial pressure (PO_2_ and PCO_2_, respectively) in the medium at the end of culture measured using the offline method. (**C**) The number of harvested cells in each bag. Data represent mean ± standard deviation (*n* = 3); *P < 0.05, **P < 0.01, n.s., not significant; #5: the values when both the top and bottom films were replaced with gas-impermeable ones.

Our microwell culture bags are made of a gas-permeable film to induce sufficient gas exchange in a closed system. Because gas exchange in the medium is performed through the film, it is possible to culture cell in a closed system. In open culture vessels, such as Petri dishes, the gas in the medium is exchanged only at the gas–liquid interface on the surface of the medium. For culture bags, it is expected that gas exchange would not only occur on the top surface but also on the bottom surface closest to the cells. However, in the current culture system, the top surface of the bag was pressed by the pressing plate of the jig, which might interfere with the gas-exchange efficiency. To assess the efficiency of gas exchange from the top or bottom of the bag during cell culture, we prepared a variety of microwell bags in which the top or bottom film was made of either a gas-permeable or -impermeable film (Fig. 2A and Supplementary S6). The permeability of O_2_ and CO_2_ measured under 80% humidity was 7.83 × 10^3^ and 3.45 × 10^4^ mL/m^2^·day·atm in the gas-permeable film and 1.32 × 10^2^ and 3.25 × 10^2^ mL/m^2^·day·atm in the gas-impermeable film, respectively (Supplementary Fig. S6).

Next, we seeded undifferentiated hiPSCs in each bag and cultured them for 2 days before measuring the oxygen partial pressure (PO_2_) and carbon dioxide partial pressure (PCO_2_) of the culture solution. In the bag with the gas-impermeable film at the bottom, the PO_2_ was the lowest and the PCO_2_ the highest among the four bags tested (Fig. 2B). In contrast, the PO_2_ was not significantly different in the other three bags—a gas-permeable film at the top and bottom either with or without a pressing plate and a gas-impermeable film only at the top. These results suggest that gas exchange between the inside and outside of the bag is more advantageous via the bottom surface, which is closer to the cells, than via the top surface. This fact possibly explains the slight difference in PCO_2_ among the tested bags. Notably, a holding plate on the top surface had little effect on gas exchange. Lastly, the cell number was identical in all the tested groups (Fig. 2C), suggesting that the difference in PO_2_ and PCO_2_ among the tested bags was attributable to the gas exchange efficiency of the bags rather than to the difference in the cell number.

### Reaggregation of iPICs

To investigate the feasibility of the microwell bag for homogeneous aggregation of iPICs, we differentiated iPICs from hiPSCs in a stirred bioreactor and cryopreserved the cells for further analysis. Immediately before freezing, 94.1% of cells expressed pancreatic endocrine cell markers (PDX1^+^CHGA^+^) and 42.4% expressed pancreatic β-cell markers (C-peptide^+^NKX6.1^+^). Only 0.2% of cells were positive for the proliferating cell marker, Ki67 (Supplementary Fig. S7). These results indicate that most of the cells were pancreatic endocrine cells with only a few proliferating cells.

**Figure 3:**
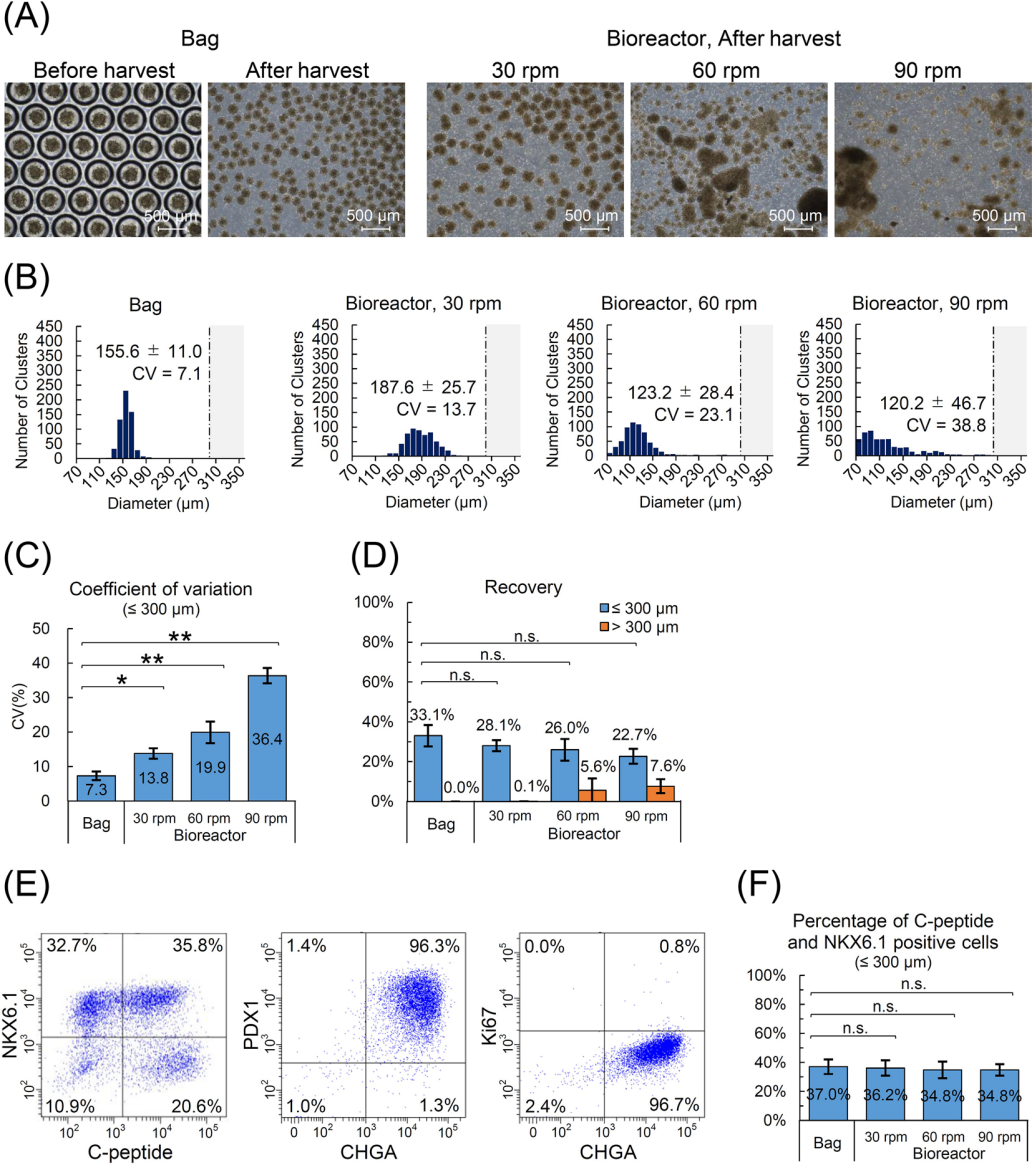
Formation of the uniform induced pluripotent stem cell-derived pancreatic islet cell (iPIC) clusters using a small-scale microwell bag. (**A**) Representative phase-contrast images of iPIC clusters. After thawing, iPICs were cultured for 4 days to form a cluster in either a φ 0.50 mm small-scale bag or bioreactors at different agitation rates of 30, 60, and 90 rpm, respectively. Scale bars = 500 μm. (**B**) Distribution of iPIC cluster size after 4-day culture. The size of the iPIC clusters was analyzed from the phase-contrast images. Clusters larger than 300 μm in diameter were excluded from the analysis (mean ± standard deviation). (**C**) Distribution of the cluster size. (**D**) Recovery rate of iPICs of ≤ 300 μm or > 300 μm in diameter. The number of cells was counted in each fraction of the group and the recovery rate was calculated relative to the number of cells seeded. (**E**) Representative dot plots of flow cytometry analyses for pancreatic endocrine cell markers and a proliferating cell marker. (**F**) Percentages of C-peptide- and NKX6.1-positive cells. Data represent mean ± standard deviation (*n* = 3); *P < 0.05, **P < 0.01, n.s., not significant.

The cryopreserved cells were thawed and seeded into a small-scale bag (φ 0.50 mm, 4 mm height) or a bioreactor for clustering and cultured for 4 days. We aimed for a cluster size, 150 μm in diameter, which is the standard size of the human islets for convenience^18^. The aggregated iPICs in the bag were visibly round and homogenous (Fig. 3A and Supplementary Video S2). In contrast, we observed many rough aggregates in the bioreactors, some of which were in the process of fusion between clusters. In addition, some enormous clusters were present in the bioreactors at rotation speeds of 60 and 90 rpm. Next, we measured the cluster sizes after excluding the over-sized clusters (300 μm in diameter as threshold). In the bioreactor, the distribution of the cluster size widened and shifted toward a smaller size as the rotational speed increased (Fig. 3B). The CV of the cluster size was 7.3 ± 1.24 in the bag but increased from 13.8 ± 1.54 to 36.4 ± 2.24 in the bioreactor as the rotational speed increased, indicating that the bag forms clusters with less variation in size compared with that in the bioreactors (Fig. 3C).

Because iPICs hardly proliferate, unlike undifferentiated iPSCs, they are not expected to increase in number during recovery culture after thawing. Therefore, obtaining a high yield of iPICs from a culture vessel is desirable. We evaluated the recovery rate with distinguishing over-sized clusters from the harvested cells, considering large cell clusters may not be suitable for implantation. Omitting the over-sized clusters, the recovery rate of the bag culture was 33.1% ± 5.3% (Fig. 3D). The recovery rates of the bioreactors were slightly lower than those of the bags and tended to decrease as the rotation speed increased, owing to the increase in the number of over-sized clusters. This finding suggests that collecting clusters from the culture bag is comparable to the collection using conventional reactors. It should be noted that the relatively low recovery rate may be attributed to the method used for the induction of differentiation, which includes the elimination of non-target cells during the reaggregation culture. After 4 days of culture in the bag, approximately 96.3% of cells were positive for pancreatic endocrine cell markers, 35.8% were positive for pancreatic β-cell markers, and few proliferating cells were Ki67-positive (Fig. 3E). However, there was no difference in the differentiation efficiency between the clustering methods (Fig. 3F and Supplementary Fig. S8).

### Large-scale production of homogeneous iPIC clusters

**Figure 4:**
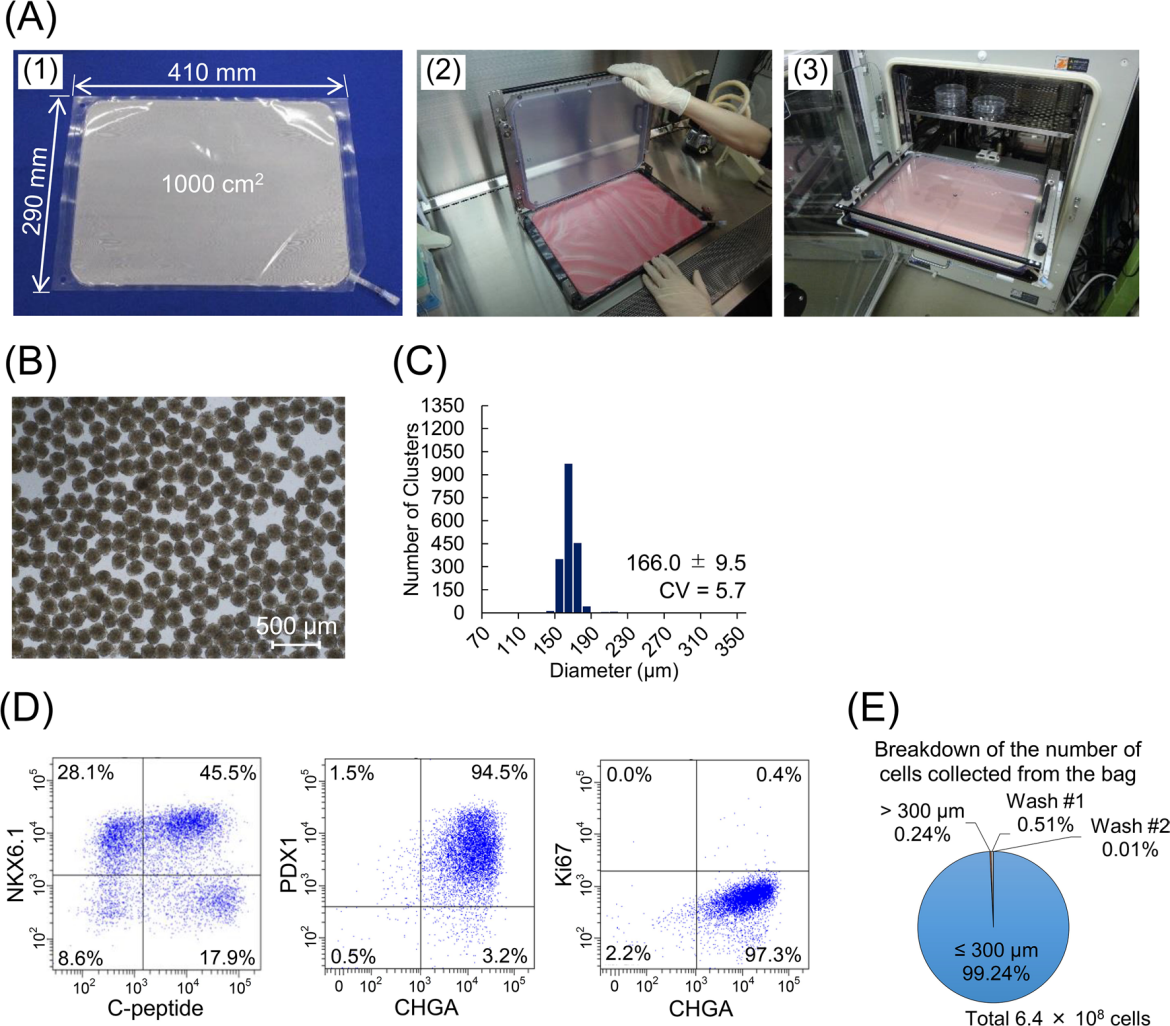
Large-scale production of the uniform-sized induced pluripotent stem cell-derived pancreatic islet cell (iPIC) clusters. (**A**) The design of a large-scale microwell bag and jig. (A-1) The appearance of a large-scale microwell bag. The bag was made of gas-permeable film with a port at one corner. The culturing area is 1000 cm^2^. (A-2) The appearance of the jig with an open cover during attachment of the large-scale microwell bag. (A-3) Placing the jig in the incubator. The shelf in the incubator has been modified for horizontal removal of the jig–bag assembly. (**B**) A representative phase-contrast image of the iPIC clusters after harvesting. After thawing, the iPICs were cultured for 4 days to form a cluster in the large-scale bag. Scale bar = 500 μm. (**C**) Distribution of iPIC cluster size after 4-day culture in the large-scale bag analyzed using phase-contrast images of the clusters (mean ± standard deviation). In total, 1800 clusters were used for the measurement. (**D**) Representative dot plots of flow cytometry analyses for pancreatic endocrine cell makers and a proliferating cell marker. (**E**) Breakdown of the number of cells collected from the bag. Clusters larger than 300 μm in diameter were separated using a cell strainer. After harvesting, the clusters remaining in the bag were washed twice with PBS and collected (Wash #1 and #2).

To obtain the number of cell clusters required for islet transplantation such that 10^5^–10^6^ islets could be obtained per bag, we designed a large-scale microwell bag (φ 0.35 mm, 4 mm height) having 6.5 × 10^5^ microwells in a culture area of 1000 cm^2^ and a corresponding holding jig (Fig. 4A). We examined the feasibility by culturing the iPICs for 4 days. Similar to the small-scale bag, uniformly shaped iPIC clusters were formed in the large-scale bag (CV = 5.7; Fig. 4B–C and Supplementary Video S3). The harvested iPICs exhibited a pancreatic endocrine phenotype, and the differentiation efficiency was similar to that in a small-scale culture (Fig. 4D), suggesting that the large-scale bag is comparable (and/or superior) to the small-scale bag for cell clustering in terms of size uniformity and cell proportion.

For operability, we measured the time required for harvesting. The iPIC clusters formed in the culture bag could be dropped from the microwells by turning the bag upside down. Thereafter, all the culture solutions drained through the port of the large-scale bag by hanging for < 8 min (Supplementary Video S4). Remarkably, even after excluding the large clusters with unsuitable size for transplantation and the clusters left in the bag, > 99% of the total number of cells after culturing could be collected (Fig. 4E). These results indicate the feasibility of operating the bag culture for handling 10^5^–10^6^ cell clusters.

## Discussion

Clustering and aggregation of adhesive cells is one of the fundamental approaches to recapitulate the in vivo microenvironment of cells in vitro and have been applied to an increasing number of cell types^8–15,19,20^. In this study, we developed novel culture bags with microwells to provide an alternative method for cell clustering. The bag culture method has many advantages over the conventionally used methods involving hard-plastic culture vessels with microwells and hanging drop methods. For uniformity, we show that a proper control of temperature during cell seeding allows the formation of uniform-sized clusters regardless of the size of the wells or the height of the medium (Fig. 1). This suggests that we can design various well sizes and bag heights (medium heights) according to the purpose of culture. For scalability, we demonstrate the feasibility of bags to produce cell clusters fomr a small scale (< 10^5^ clusters) to a large scale (10^5^–10^6^ clusters) by culturing cells in bags with microwells at the bottom (Fig. 4). The bag has a semi-closed structure, and is simply applied to a closed culture system. In addition, the bag exhibits user-friendly operability: (1) microbubbles trapped in each microwell could be removed by installing the bag with medium into the jig and leaving overnight; (2) clusters could be detached from the microwells by simply turning the bag upside down; and (3) almost all clusters (> 99%) could be collected by simply hanging the bag (Supplementary Fig. S2, Supplementary Videos S1 and S4, Fig. 4E). Moreover, the jig allows carrying the bag without the risk of clusters spilling to other wells.

We demonstrate the feasibility of using the microwell bag to produce 10^5^–10^6^ uniform-sized clusters in the process of iPIC reaggregation after thawing, aiming to obtain the the cell number currently required for islet transplantation. The CV values indicate that the developed bag maintained high uniformity for cluster formation even at this scale, which is comparable to the level obtained at a small scale (< 10^5^ clusters). Although it is possible to produce 10^5^–10^6^ clusters using a bioreactor, the generated clusters are relatively heterogeneous in size^21–23^. In fact, we found that the size variation decreased as the stirring speed decreased, whereas unintended huge cell clusters were formed even at 30 rpm^13^, in contrast to the result obtained with the static bag culture. Higher size variation in stirring culture compared with static culture potentially arises from the frailty and increased susceptibility of cells immediately after thawing^24^. We assume that genomic DNA eluted from dead cells entangles clusters to form oversized clusters^25^. Differences in the size of clusters during culture can affect the quality and quantity of the obtained cells^14,15^, likely owing to the different microenvironments for individual cells. Although glucose-stimulated insulin secretion is one of the important functions of islets, we did not evaluate it because this study focused on homogeneous size aggregate formation, but we hope to consider it in the future. In addition, during the early phase after implantation, the oxygen and nutrient supply depends on diffusion, whereas the size of the cluster limits the supply^26^, especially in the case of encapsulation in an immuno-isolative device^27,28^ that does not allow the formation of blood vessels in the proximity of cells. Therefore, for applications requiring high uniformity in the cluster size, a static method, such as the bag culture developed in this study, could be a suitable option.

The gas in the medium in open culture vessels, such as Petri dishes, is exchanged only at the gas–liquid interface on the surface of the medium. In contrast, we demonstrate that gas exchanges in the bags occur not only at the top surface but also at the bottom surface closest to the cells. Therefore, the gas exchange through the bag is considered to be sufficient and/or superior to conventional plastic vessels^29^, despite the semi-closed structure. Gas exchange may be improved by using a gas-permeable plate or a hanging drop and culturing at the air–liquid interface; however, these methods are difficult to operate, and clusters are easily disturbed (moved) when handling the vessels. The bags developed in this study solve this issue by applying an air-phase-free approach together with the added support from the jig, representing a feasible alternative. In this study, we did not find any advantages in gas exchange near culturing cells evaluated by the proliferation of undifferentiated hiPSCs (Fig. 2C). However, it might be effective for different cell types that require high oxygen consumption, such as large cell clusters, organoids, or when the depth of the medium results in exceptionally long distances between the cells and the air-phase.

The microwell bag culture can be applied in the production of clusters formed from cell types other than the PSCs and iPICs used in the current study. For instance, in cell therapies using adhesive cells, such as mesenchymal stem cells, the cell clusters showed better engraftment than single-cell suspensions^30–32^. Furthermore, it can be applied in preparing cells for differentiation, such as the serum-free culture of the embryoid body-like aggregate method for neural cells^33^. Lastly, it could also be used in restructuring organoids, such as liver organoids, by mixing hepatocytes, endothelial cells, and mesenchymal cells^15^, or kidney organoids, by mixing nephron progenitor cells and ureteric bud cells^34^. In conclusion, the technology developed in this study to produce a large number of uniform clusters is expected to be applied for the preparation of cell clusters used in transplantation therapy and organoids for screening in regenerative medicine.

## Materials and methods

### hiPSCs

Two hiPSC lines, 1231A3^35^ and Ff-I14s04, were obtained from the Center for iPS Cell Research and Application of Kyoto University (Kyoto, Japan). Ff-I14s04, an HLA homozygous iPSC line, was established as previously described^35^. The use of the iPSC lines was approved by the ethical review committee of Shonan Health Innovation Park (Fujisawa, Kanagawa, Japan) and Kyoto University. For the 1231A3 line, cells were maintained according to the protocol issued by the Center for iPS Cell Research and Application (CiRA, Kyoto University). Briefly, we cultured the cells on an iMatrix511™ (Nippi, Tokyo, Japan)-coated culture surface using Stem Fit® AK02N (Ajinomoto, Tokyo, Japan). We incubated the cells at 37 °C, 95% humidity, and 5% CO_2_ atmosphere unless stated otherwise. Every 7 days, the cells were passaged by treatment with TrypLE™ (Thermo Fisher Scientific, Waltham, MA, USA). For the Ff-I14s04 line, we maintained the cells in the StemFit AK03N medium (Ajinomoto) and passaged them using EDTA (0.5 mM) twice weekly.

### Design of microwell bag and jig

We developed the membranes for the cell culturing bag using our original films. The gas-permeable membrane was made of a 110 μm-thick linear low-density polyethylene (LLDPE) film. The gas-impermeable membrane was made of a multilayer film consisting of three layers: LLPDE, gas-impermeable ethylene–vinyl alcohol copolymer (EVOH), and adhesive resin (AD). The thickness of the gas-impermeable membrane was 118 μm, and the ratio of the film thickness was 57:15:23 (LLDPE:AD:EVOH) (Supplementary Fig. S6). We measured gas permeability using a gas/water vapor permeation analysis system (GTR-10X; GTR TEC, Kyoto, Japan) (Supplementary Fig. S6).

For the lower membrane, hemispherical microwells were thermoformed in a staggered pattern in a 50 cm^2^ hexagonal cell culturing area of the film. The diameter and number of the microwells are shown in Supplementary Fig. S3. For an upper membrane as a top cover, a vertical wall was thermoformed along the edge of the culture area to provide space for the medium. The height of the vertical wall, defined as the bag height, was designed as 2, 4, 6, and 8 mm for medium volumes of 10, 20, 30, and 40 mL, respectively. A port for injecting or draining the culture solution into/from the bag was placed in the center of the short side of the rectangular bag. The culture area is hexagonal in consideration of the ease of draining the culture solution from the port and future expandability, such as the installation of multiple ports. The upper and lower membranes and port components were heat-welded around the hexagonal culture area to form a bag. At the tip of the port, a Needleless Injection Site (#2901280167; Qosina, Ronkonkoma, NY, USA) to connect a syringe was attached via a short tube. When using the gas-impermeable membranes, the inner surface side in contact with the cells was designated as the LLDPE layer. We coated the inner culture surface with 2-methacryloyloxyethyl phosphorylcholine polymer (LIPIDURE®; NOF, Tokyo, Japan) to inhibit cell adhesion.

To increase the usability of the culture bag, we developed a jig composed of three parts: a base plate on which the culture bag was placed, a pressing plate that pressed the culture bag from above, and a cover that supported the pressing plate (Supplementary Fig. S1). The pressure applied to the bag by the pressing plate was set to 200–400 kgf/m^2^. A part of the base plate and the pressing plate was transparent, through which the culture bag contents could be observed without detachment from the jig. In addition, the external dimensions of the jig are of the SBS standard size of 128 mm × 86 mm, allowing operation similar to that of commercially available culture plates.

### Small-scale bag culture of undifferentiated iPSCs

A day before cell seeding, the bag was half-filled with medium, attached to the jig, and incubated at 37 °C, 95% humidity, and 5% CO_2_ atmosphere for > 18 h to remove the microbubbles caught in each microwell. When using the gas-impermeable membranes, the microbubbles were floated by gently tapping outside the bag, and the floated bubbles were sucked out through the port with a syringe. The next day, cells for seeding were dissociated into single cells and centrifuged at 150×*g* for 2 min; thereafter, the supernatant was removed to obtain a cell pellet. The cells were suspended at a ratio of 250 cells per microwell in a medium at 37 °C. Before cell seeding, the bag was detached from the jig, which remained at 37 °C, and cell slurry was injected into the bag, followed by removal of air with a syringe. After mixing by gentle inversion, the bag was placed on a hot plate (HP-4530 N; AS ONE Corporation, Osaka, Japan) set at the target temperature. The culture solution in the bag was kept warm while mixing by applying palm pressure onto the bag from above. The bag containing the cells was reattached to the jig in the incubator and incubated at 37 °C for 2 days.

### Differentiation of iPSCs into iPICs

The hiPSCs (Ff-I14-s04) were differentiated into iPICs using a previously reported method^36^, with some modifications, as shown in Supplementary Table S1. Dissociated undifferentiated iPSCs were resuspended at a density of 2.0 × 10^5^ cells/mL cells using AK03N containing 10 μM Y-27632 (FUJIFILM Wako) and seeded into a 100 mL bioreactor (ABLE Corporation, Tokyo, Japan). The agitation rate was set to 55 rpm using Bio Jr.8 (ABLE). The cells were cultured for 24 h to induce aggregation. The culture medium was changed according to the following schedule.

Stage 1 (3 days): DMEM (#10569010; Thermo Fisher) containing 1 × B27® supplement (#17504001 or A1895601; Thermo Fisher), 1% DMSO (FUJIFILM Wako), 0.1% Pluronic® F68 (Sigma-Aldrich, Saint Louis, MO, USA), activin A (10 ng/mL; PeproTech, Cranbury, NJ, USA), and CHIR99021 (3 μM; Axon Medchem, Reston, VA, USA). CHIR99021 was added to the culture medium only on the first day.

Stage 2 (4 days): MCDB131 medium (#10372019; Thermo Fisher) containing 0.5 × B27, glucose (final concentration 10 mM; FUJIFILM Wako), NaHCO_3_ (1.5 g/L; FUJIFILM Wako), GlutaMAX™ (2 mM; Thermo Fisher), ascorbic acid phosphate magnesium salt (PMS) (58 mg/L; FUJIFILM Wako) and keratinocyte growth factor (KGF) (50 ng/mL; R&D Systems, Minneapolis, MN, USA), and 0.1% Pluronic® F68.

Stage 3 (3 days): improved MEM (#10373017, Thermo Fisher) containing 0.5 × B27, ascorbic acid PMS (58 mg/L), KGF (50 ng/mL), LDN-193189 (100 nM; MedChemExpress, Monmouth Junction, NJ, USA), 3-keto-N-(aminoethyl-aminocaproyl-dihydrocinnamoyl)cyclopamine (0.5 μM; Toronto Research Chemicals, Toronto, Canada), 4-[(E)-2-(5,6,7,8-tetrahydro-5,5,8,8-tetramethyl-2-naphthalenyl)-1-propenyl]benzoic acid (TTNPB) (10 nM; Santa Cruz Biotechnology, Dallas, TX, USA), and 0.1% Pluronic® F68.

Stage 4 (4 days): improved MEM containing 0.5 × B27, ascorbic acid PMS (58 mg/L), KGF (100 ng/mL), epidermal growth factor (50 ng/mL; R&D Systems), nicotinamide (10 mM; Stemcell Technologies, Vancouver, Canada), phorbol 12,13-dibutyrate (0.5 μM; Sigma-Aldrich), activin A (5 ng/mL), and 0.1% Pluronic® F68.

Stage 5 (2 days): MCDB131 medium containing 0.5 × B27, glucose (final concentration 20 mM), NaHCO_3_ (1.5 g/L), 1:100 GlutaMAX™, SANT-1 (0.25 μM; Sigma-Aldrich), TTNPB (10 nM), activin receptor-like kinase 5 inhibitor II (ALK5i II) (10 μM; Santa Cruz), LDN-193189 (100 nM), triiodothyronine (T3) (1 μM; Sigma-Aldrich), basic fibroblast growth factor (50 ng/mL; PeproTech), XAV939 (1 μM; Sigma-Aldrich), Y-27632 (10 μM), and 0.1% Pluronic® F68.

Stage 6 (7 days): MCDB131 medium containing 0.5 × B27, glucose (final concentration 20 mM), NaHCO_3_ (1.5 g/L), 1:100 GlutaMAX™, ZnSO_4_ (10 μM; Sigma-Aldrich), heparin sodium salt (1.4 IU/mL; Nacalai Tesque, Kyoto, Japan), RO4929097 (1 μM; Selleck Chemicals, Houston, TX, USA), ALK5i II (10 μM), LDN-193189 (100 nM), T3 (1 μM), PD-166866 (1 μM; Sigma-Aldrich), and 0.1% Pluronic® F68. PD-166866 was added to the culture medium only for the last 3 days.

After 7 days of culture of stage 6, the cell clusters were collected and treated with TrypLE™ to dissociate into single cells. The cells were cryopreserved using Bambanker® hRM (GC Lymphotec, Tokyo, Japan) and stored at –150 °C until use.

### iPIC cluster formation after cryopreservation in a small-scale bag and bioreactors

A day before cell seeding, 10 mL 2 × differentiation medium was added into a φ 0.5 mm and 4 mm height microwell bag and incubated at 37 °C to remove microbubbles (Supplementary Fig. S2). The 2 × differentiation medium was formulated as the Stage 7 medium: MCDB131 medium containing 2% fat-free bovine serum albumin (BSA) (FUJIFILM Wako), glucose (final concentration 20 mM), NaHCO_3_ (1.5 g/L), GlutaMAX™ (2 mM), 0.5 × ITS-X, ALK5i II (20 μM), T3 (2 μM), ZnSO_4_ (20 μM), heparin sodium salt (2.8 IU/mL), N-acetyl cysteine (2 mM; Sigma-Aldrich), Trolox (20 μM; FUJIFILM Wako), R428 (4 μM; Selleck Chemicals), TR05991851 (6 μM; Takeda original multi-kinase inhibitor), and Y-27632 (10 μM).

iPICs were thawed in a 37 °C water bath. The cells were suspended in improved MEM containing 0.5 × B27 and Y-27632 (10 μM). The cells were centrifuged at 350×*g* for 5 min and then resuspended in MCDB131 medium containing 2% fat-free BSA, glucose (final concentration 20 mM), NaHCO_3_ (1.5 g/L), GlutaMAX™ (2 mM), 0.5 × ITS-X, and Y-27632 (10 μM) to a density of 2.7 × 10^6^ cells/mL. Cell suspension (10 mL) was added to the prepared bag. The number of iPICs seeded in the bag was 2.7 × 10^7^ cells/mL (1500 cells/microwell), and the medium volume was 20 mL. The cells were seeded in a 30 mL bioreactor (ABLE) using a 1 × differentiation medium. The number of iPICs seeded in the bioreactor was 3.66 × 10^7^ cells, and the medium volume was 15 mL. The agitation rate of the bioreactors was set to 30, 60, or 90 rpm. The cells were incubated at 37 °C for 4 days. Reaggregation experiment was repeated three times using the same batch of cells that were cryopreserved.

### Scale-up of bag culture

A large-scale culture bag was designed at a 1000 cm^2^ rectangular culture area and height of 4 mm. A total of 6.5 × 10^5^ microwells, with a diameter size of 350 μm, were thermoformed on the lower portion of the bag. The port was placed at one of the vertices of the rectangle for easy drainage. The large-scale bag was made of the same material as the small-scale bags. A jig was also designed to fit the size of the large-scale culture bag.

The iPICs were seeded into a large-scale bag using the same method as for the small-scale bag above. Briefly, a day before cell seeding, the bag was half-filled with 2 × differentiation medium and incubated at 37 °C to remove the microbubbles in the microwells (Supplementary Fig. S2). After thawing, the cells were seeded into the bag placed on a hot plate at 37 °C. The number of seeded cells was 9.75 × 10^8^ cells (1500 cells/microwell), and 200 mL medium was added to a total volume of 400 mL. The bag was then incubated with the jig at 37 °C for 4 days.

### Imaging clusters, cell counting, and medium gas analysis

Clusters in microwells were observed using a phase-contrast microscope (CKX53; Olympus Corporation, Tokyo Japan) with a UPlanFL N × 4 objective lens (NA 0.13; Olympus Corporation) and imaged using a CMOS camera L-835 (Hozan Tool Industrial, Osaka, Japan) or a CCD camera DP27 (Olympus Corporation).

Before collecting the clusters, a portion of the medium was sampled from the bag, and PO_2_ and PCO_2_ in the medium were measured using i-STAT® 300F (Abbott Laboratories, Chicago, IL, USA).

To collect the clusters from the bag, the bag was detached from the jig and placed upside down to float clusters from inside the microwells. After inflating the bag by injecting air through the port with a syringe (20 mL for a small bag, 400 mL for a large bag), the entire culture solution was collected with the syringe. For culturing in the bioreactors, cells were directly sucked up from the vessel with a serological pipette. For large-scale culture bags, the bag was hung on a stand to drain all the culture solution through a tube connected to the port and collected in a centrifuge tube under gravity. At this time, large clusters over 300 μm in diameter were removed using a cell strainer (pluriSelect Life Science, Leipzig, Germany) (Supplementary Video S4).

The harvested culture solution was transferred to a 10 cm dish (from the small bags or bioreactors) or a T-225 Flask (from the large bag), and phase-contrast images were obtained for analysis of the shape and diameter of the clusters.

After taking images on a 10 cm dish, the iPIC clusters were divided into two groups using a cell strainer—those with a diameter of 300 μm or less and those with a diameter > 300 μm. Aliquots of the collected clusters were dissociated into single cells by treating with TrypLE™. The cell number was counted using a hemocytometer (One cell Counter; One Cell, Hiroshima, Japan) for undifferentiated iPSCs and by using a NucleoCounter® NC 200™ (ChemoMetec A/S, Allerod, Denmark) for iPICs. In addition, the inside of the large bag after draining the culture solution was washed twice with 50 mL PBS (FUJIFILM Wako), and the clusters remaining in the bag were collected for cell counting. After cell counting, the cells were fixed and immunochemically stained for flow cytometry analysis.

### Cluster size analysis

The size of the cell clusters was measured from the phase-contrast images. For the small-scale bag, the culturing area was divided into 55 compartments with ~ 1 cm^2^ area to analyze the relationship between the position inside the bag and the cluster size (Supplementary Fig. S1). One image of the center of each compartment was taken. The field of view of one image was 2.88 mm × 2.16 mm. The diameters of the clusters were measured using the image processing software, WinROOF™ (MITANI Corporation, Tokyo, Japan). The outline of a cell cluster was extracted, and the diameter of the circle corresponding to the projected area was defined as the diameter of the cluster. The average diameter was calculated from all the clusters shown in an image.

To investigate the size distribution of the collected clusters, the entire harvested culture solution was transferred to a 10 cm dish or a T-225 flask, and images of the clusters dispersed and settled on the bottom surface were obtained. Multiple images were taken while repositioning so that the areas did not overlap. The field of view of one image was 2.88 mm × 2.16 mm for undifferentiated iPSCs and 3.35 mm × 2.63 mm for iPICs. The diameter of the clusters was measured using WinROOF™. The diameters of 600 clusters from each small bag and the bioreactor were measured. For the large bag, the 600-cluster measurement was repeated three times to obtain a value for 1800 clusters.

### Flow cytometry analysis

Cells were fixed and permeabilized with BD Cytofix/Cytoperm™ (BD Corporation, Franklin Lakes, NJ, USA). The cells were stained for various intracellular markers for analysis on a BD LSRFortessa™ X20^37^. The primary antibodies used in this study are listed in Supplementary Table S2.

### Data analysis and statistics

The Dunnett’s multiple-comparison test was performed at a statistical significance level of *P* < 0.05. All statistical analyses were performed using the Statistical Analysis System v9.3 (SAS Institute, Cary, NC, USA).

## Supplementary Information

Supplementary Video 1.

Supplementary Video 2.

Supplementary Video 3.

Supplementary Video 4.

Supplementary Information 1.

## Data Availability

The datasets generated during and/or analysed during the current study are available from the corresponding author on reasonable request.
