# Supplementary material for: Microwell bag culture for large-scale production of homogeneous islet-like clusters
Source: Sci Rep. 2022 Mar 25;12:5221. doi: 10.1038/s41598-022-09124-w (PMC8956638; doi:10.1038/s41598-022-09124-w)
Supplement: Supplementary file 5 — Supplementary Information 1. [file 41598_2022_9124_MOESM5_ESM.pdf]

Supplementary Information

Microwell bag culture for large-scale production of homogeneous islet-like clusters

Ryo Suenaga, Shuhei Konagaya, Junji Yamaura, Ryo Ito, Satoshi Tanaka, Yoichi Ishizaki, and Taro Toyoda

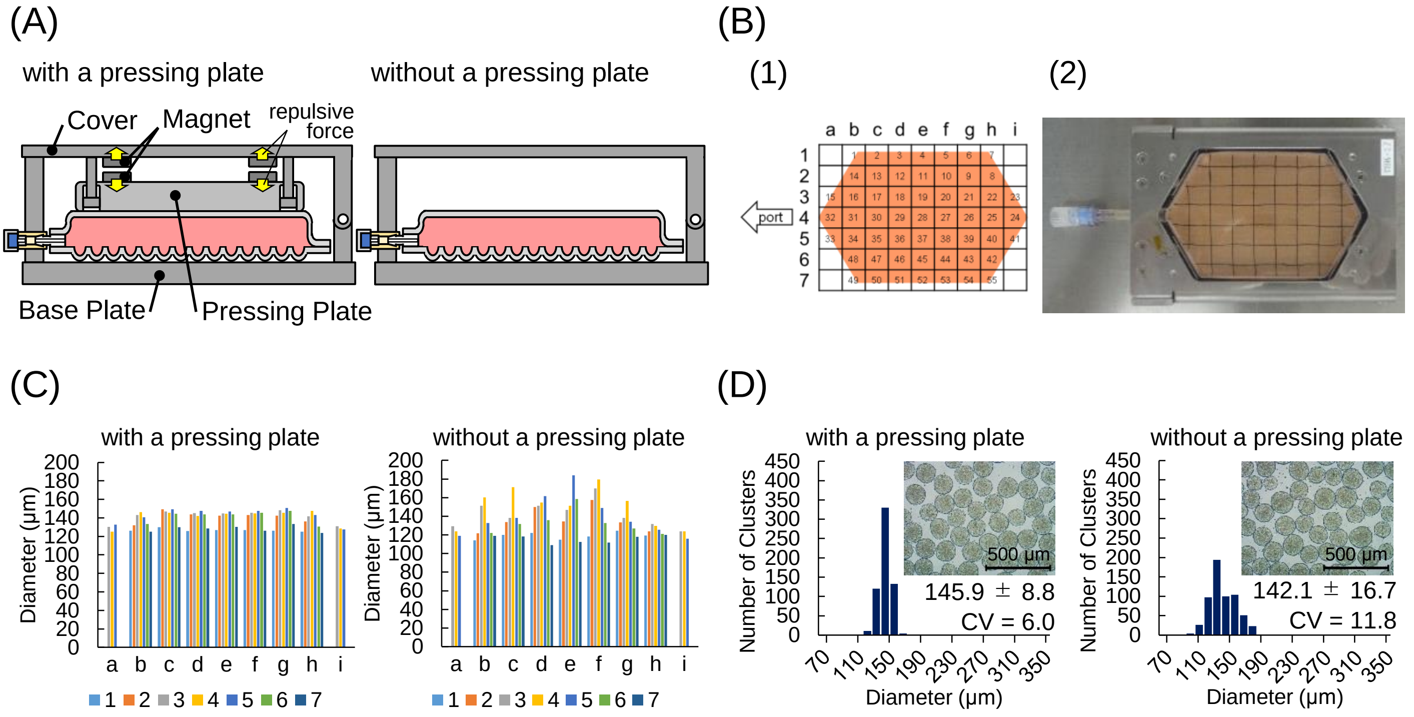

Supplementary Figure S1. Improvement of cluster size uniformity using a jig. Human induced pluripotent stem cells (hiPSCs) were cultured for 2 days in the microwell bags attached to the jigs with or without a pressing plate. (A) Schematic cross-sectional diagrams of microwell bag–jig assembly with or without a pressing plate. Four pairs of magnets that repel each other were placed on the upper surface of the pressing plate and the inner surface of the cover. The force of the magnet kept the upper and lower surfaces of the bag parallel. (B) The culture area in the microwell bag was divided into 55 compartments by squares of approximately 10 mm sides and labeled as indicated for subsequent analysis. (B-1) The schematic diagram of compartments. (B-2) A photo of a microwell bag with compartment lines drawn and attached to the jig. (C) Individual size of the hiPSC clusters 2 days after culturing in each compartment. 1-7 and a-i indicate the analyzed positions, as illustrated in (B-1). Detailed information is provided in Materials and Methods. (D) Distribution of hiPSC cluster sizes harvested from the bag. The size of the clusters was analyzed from their phase-contrast images (mean ± standard deviation; CV: coefficient of variation). A total of 600 clusters were used for the measurement. Scale bar = 500 µm.

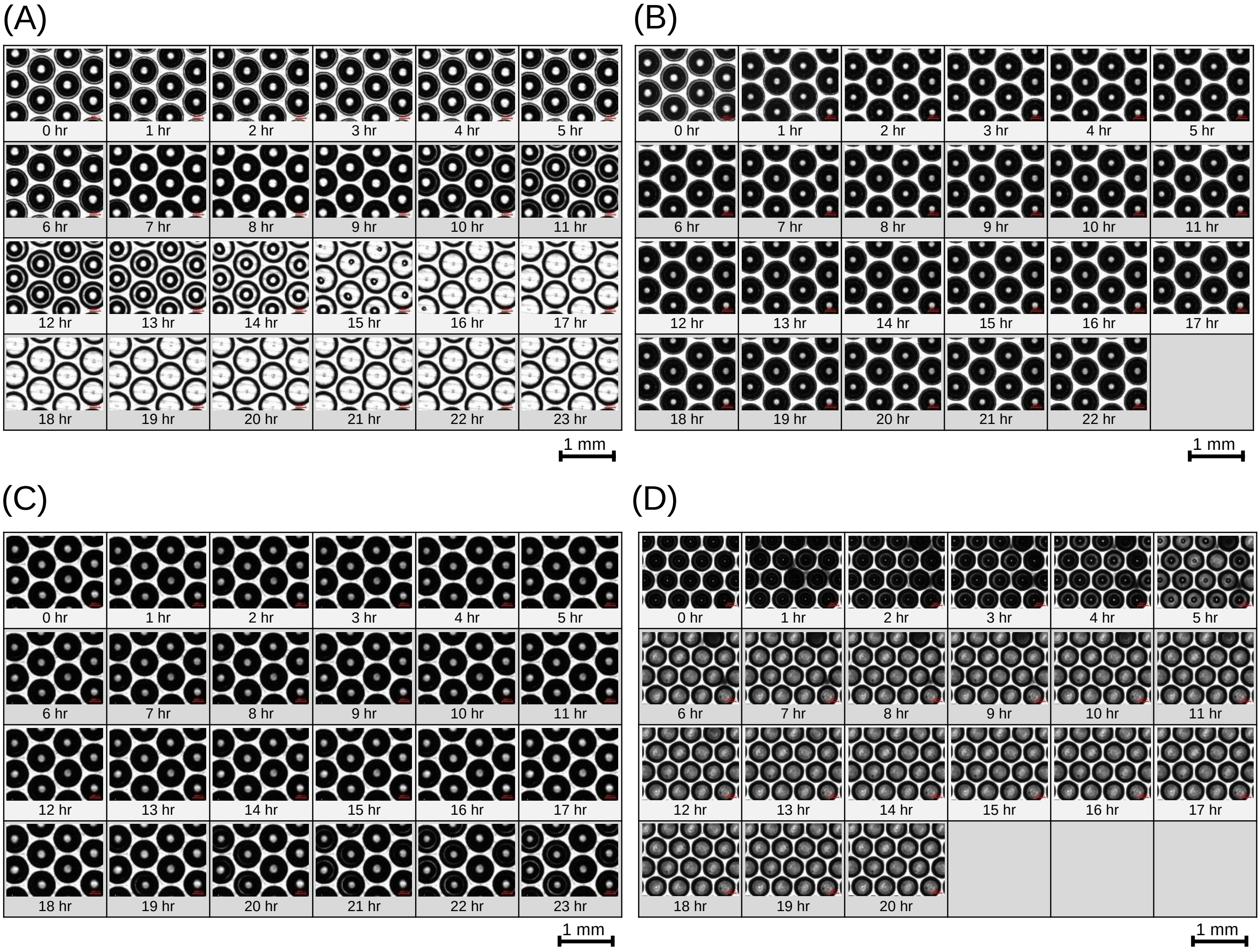

Supplementary Figure S2. Removal of air bubbles trapped in each microwell. Time-course images of the microwell bags. The bags were filled with the culture medium, attached to a jig with or without applying pressure, and incubated at 37 ° C for 24 h. When no pressure was applied to the bag, the pressing plate was removed from the jig. (A) A gas-permeable small-scale bag with pressure applied. (B) A gas-permeable small-scale bag without applying pressure. (C) A gas-impermeable small-scale bag with pressure applied. (D) A gas-permeable large-scale bag with pressure applied. Scale bars = 1 mm.

(A)

Diameter and number of microwells in small-scale bag					
Diameter (mm)	0.35	0.50	0.70	0.87	1.26
Number	32,000	18,000	9,600	6,300	3,100

(B)

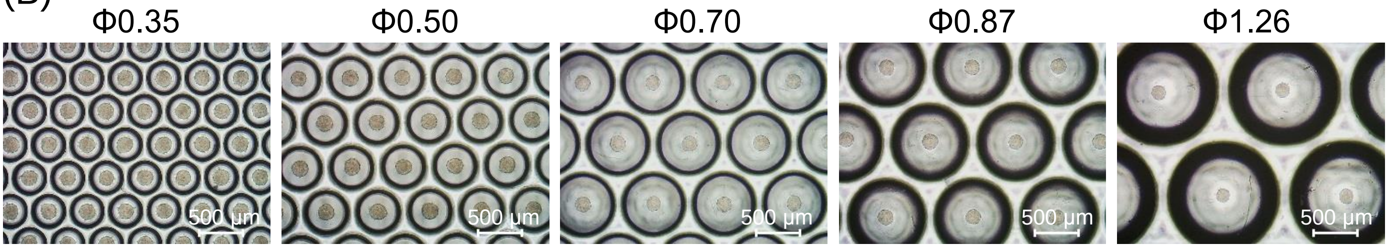

Supplementary Figure S3. Diameter and number of microwells in small-scale bags. (A) A summary of the type of microwell bags used. (B) Phase-contrast images of human induced pluripotent stem cell (hiPSC) clusters 2 days after culturing. The images correspond to Fig. 1C. Scale bar = 500 μm.

(A)

Bag height: 2 mm<br>Medium: 10 mL	Bag height: 4 mm<br>Medium: 20 mL	Bag height: 6 mm<br>Medium: 30 mL	Bag height: 8 mm<br>Medium: 40 mL

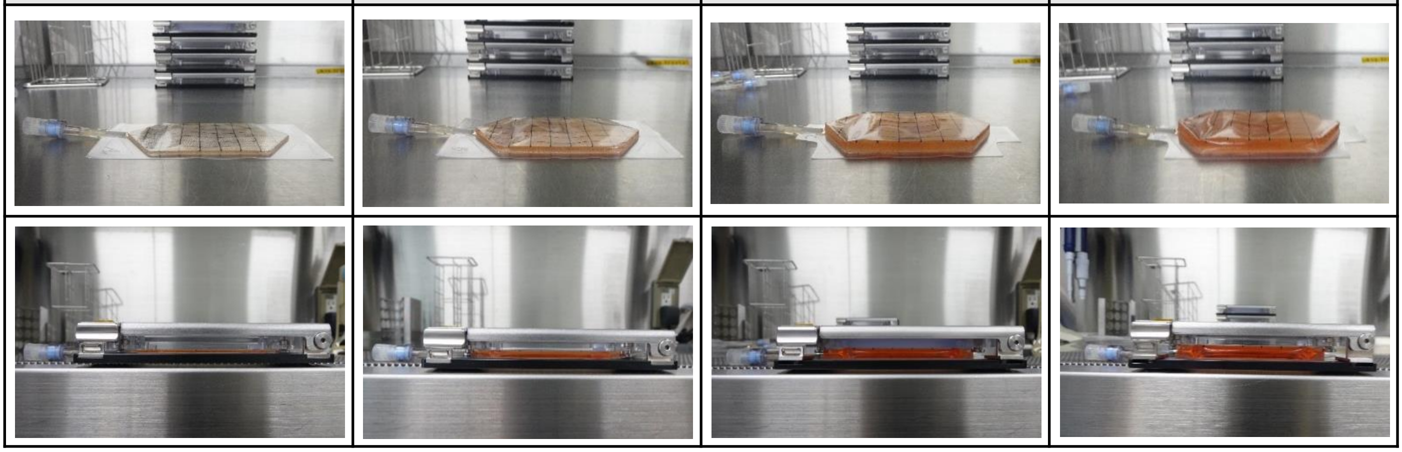

(B)

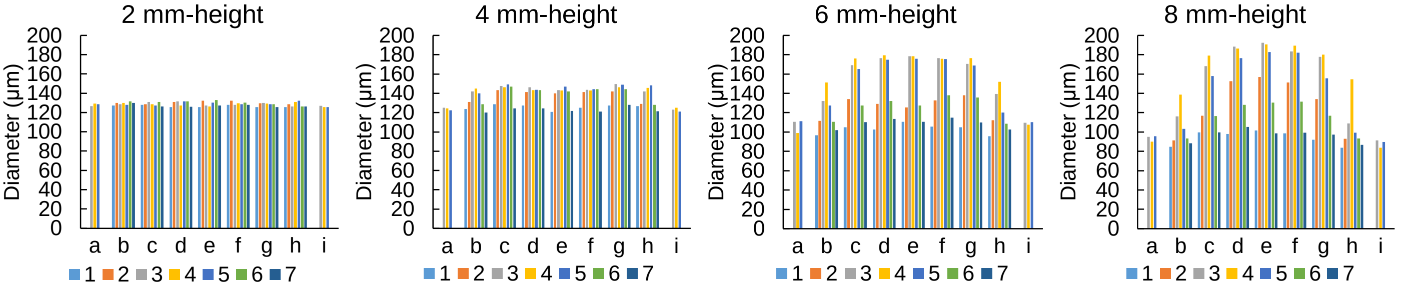

Supplementary Figure S4. The height of bags affects the uniformity of clusters. (A) Photographs of microwell bags with heights of 2, 4, 6, and 8 mm, respectively, filled with the corresponding amount of medium with (lower panels) or without jig assembly (upper panels). (B) Individual size of human induced pluripotent stem cell (hiPSC) clusters 2 days after culturing in each compartment. The grid labeling is depicted in Supplementary Fig. 1B.

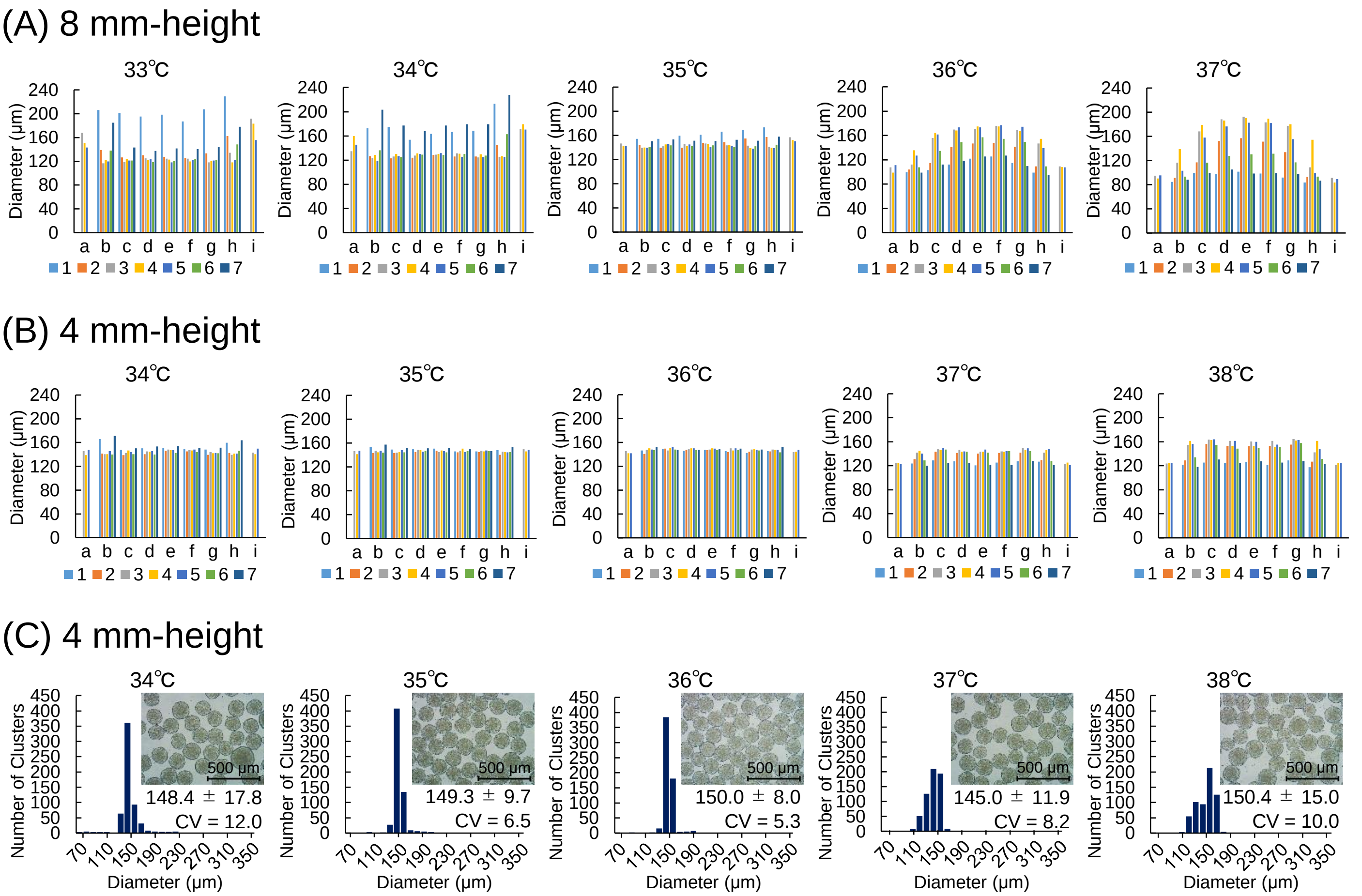

Supplementary Figure S5. Optimizing the temperature of culture medium at cell seeding improves uniformity of cell clusters. Size of human induced pluripotent stem cell (hiPSC) clusters 2 days after culturing. (A–B) Individual size of hiPSC clusters 2 days after culturing in each compartment. The grid labeling is depicted in Supplementary Fig. 1B. hiPSCs were seeded in an 8 mm (A) or 4 mm height (B) bag at different temperatures. (C) Distribution of hiPSC cluster sizes harvested from the 4 mm height bags. The cluster size was analyzed from the phase-contrast images of the clusters (mean ± standard deviation). A total of 600 clusters were used for the measurement. The results at 37 ° C correspond to the 4 mm height bag in Fig. 1D. Scale bar = 500 μm.

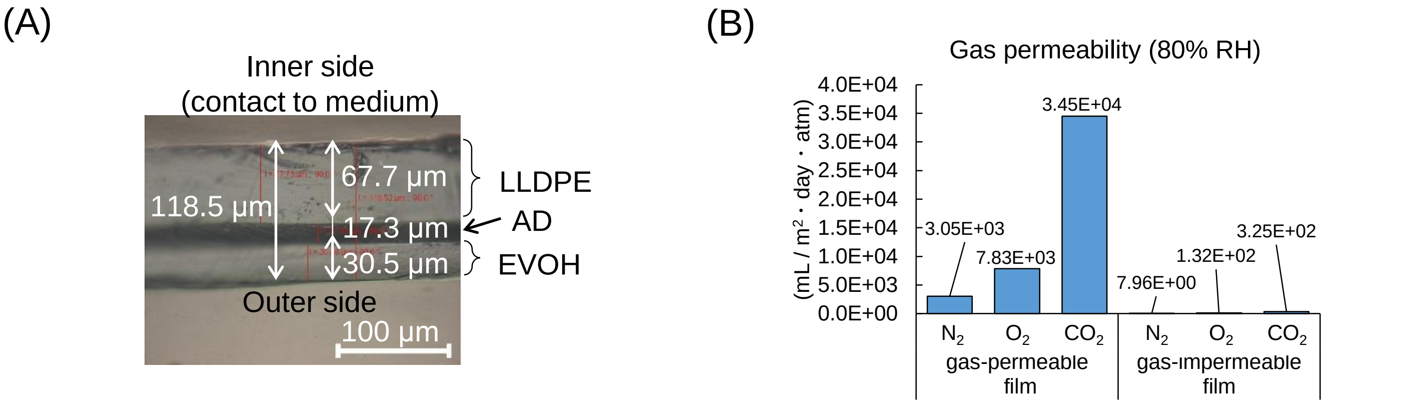

Supplementary Figure S6. Specifications of gas-impermeable film. (A) A cross-sectional image of gas-impermeable film. Scale bar = 100 μm. (B) The nitrogen, oxygen, and carbon dioxide permeability of the gas-permeable and -impermeable films. Gas permeation was measured under 80% relative humidity.

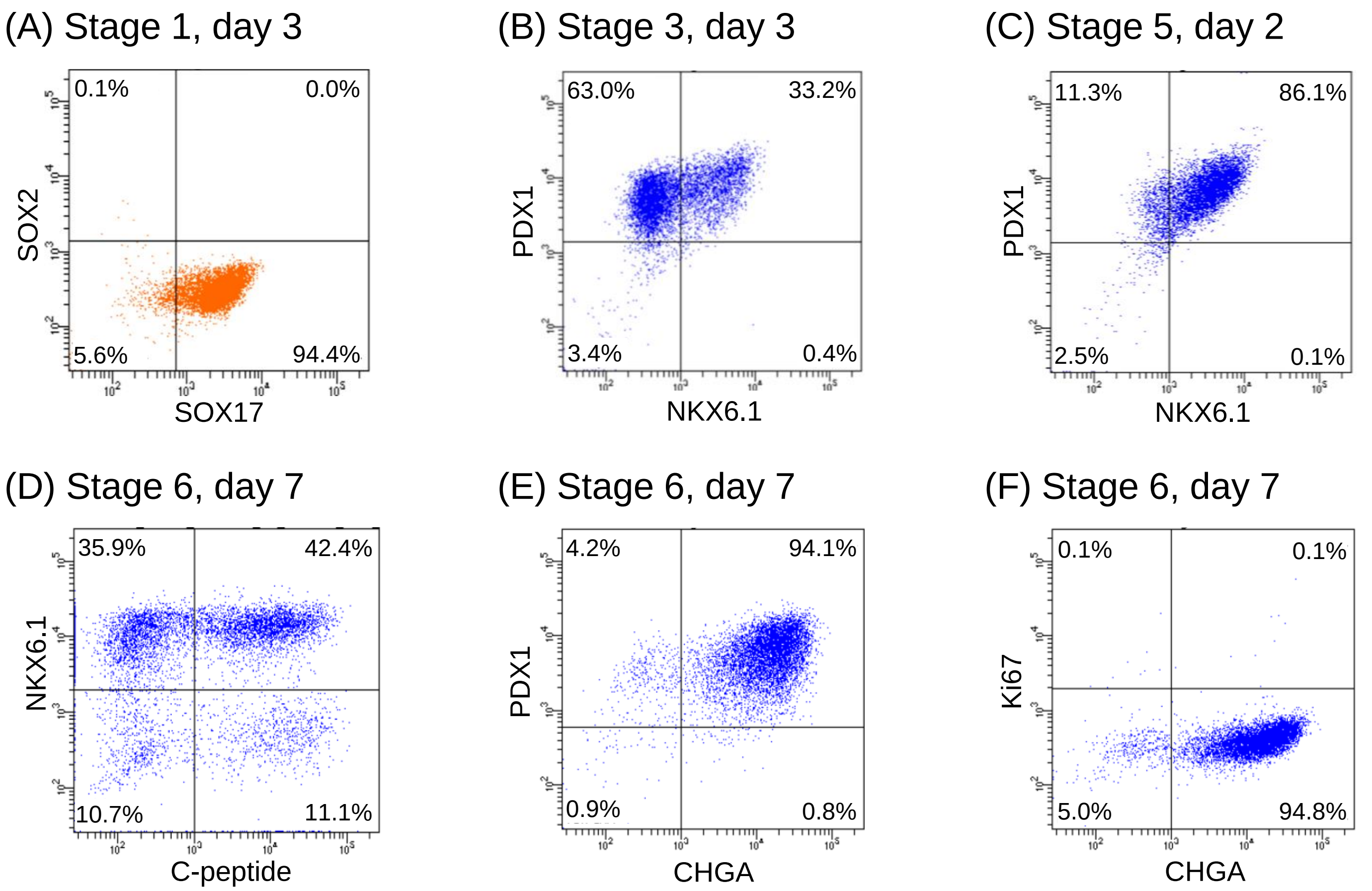

Supplementary Figure S7. Differentiation of human induced pluripotent stem cells (hiPSCs) into induced pluripotent stem cell-derived pancreatic islet cells (iPICs). (A–E) Dot plots of flow cytometry analyses for differentiation stage markers. (A) At stage 1 day 3, cells were stained with antibodies targeting an endoderm marker, SOX17, and an undifferentiated cell marker, SOX2. (B–C) At stage 3 day 3 and stage 5 day 2, cells were stained with antibodies targeting pancreatic endoderm markers. (D–F) At stage 6 day 7, cells were stained with antibodies targeting pancreatic endocrine cell markers and a proliferating cell marker.

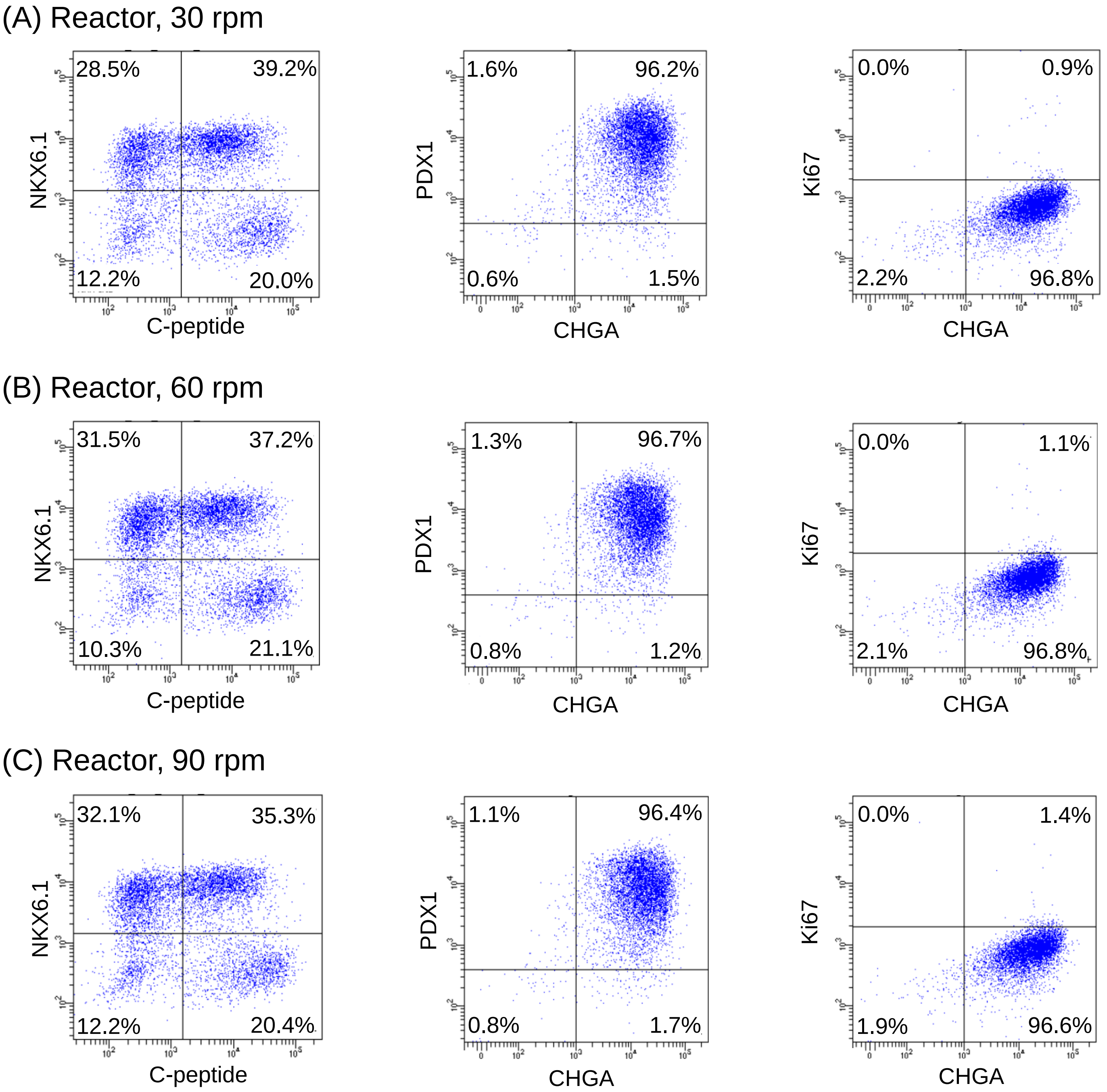

Supplementary Figure S8. Differentiation efficiency of induced pluripotent stem cell-derived pancreatic islet cell (iPIC) clusters using bioreactors. Representative dot plots of flow cytometry analyses for pancreatic endocrine cell markers and a proliferating cell marker. Cells were cultured in bioreactors at agitation rates of (A) 30, (B) 60, or (C) 90 rpm.

Supplementary Table S1. Formulation of differentiation medium.

Differentiation stage		Stage 1, day 0–1	Stage 1, day 2–3	Stage 2	Stage 3	Stage 4	Stage 5	Stage 6, day 0–3	Stage 6, day 4–7	Stage 7
Culture period	1 day	1 day	2 days	4 days	3 days	4 days	2 days	4 days	3 days	4 days
Base medium	StemFit	DMEM<br>P/S	DMEM<br>P/S	MCDB131<br>P/S<br>GlutaMAX<br>Glucose<br>$NaHCO_3$	iMEM<br>P/S	iMEM<br>P/S	MCDB131<br>P/S<br>GlutaMAX<br>Glucose<br>$NaHCO_3$	MCDB131<br>P/S<br>GlutaMAX<br>Glucose<br>$NaHCO_3$	MCDB131<br>P/S<br>GlutaMAX<br>Glucose<br>$NaHCO_3$	MCDB131<br>P/S<br>GlutaMAX<br>Glucose<br>$NaHCO_3$
Additive	Y	B27<br>Activin A<br>CHIR<br>DMSO<br>PF68	B27 INS (–)<br>Activin A<br>DMSO<br>PF68	B27<br>KGF<br>VC PMS<br>PF68	B27<br>KGF<br>LDN<br>K-CYC<br>TTNPB<br>VC PMS<br>PF68	B27<br>KGF<br>EGF<br>NAM<br>VC PMS<br>PDBu<br>Activin A<br>PF68	B27<br>SANT<br>TTNPB<br>ALK5i II<br>LDN<br>T3<br>bFGF<br>XAV<br>Y<br>VC PMS<br>PF68	B27<br>RO<br>ALK5i II<br>LDN<br>T3<br>VC PMS<br>PF68<br>Heparin<br>$ZnSO_4$	B27<br>RO<br>ALK5i II<br>LDN<br>T3<br>VC PMS<br>PD<br>PF68<br>Heparin<br>$ZnSO_4$	BSA<br>ITS-X<br>ALK5i II<br>Trolox<br>R428<br>PD<br>TR<br>Y<br>T3<br>NAC<br>Heparin<br>$ZnSO_4$

The formulation of differentiation medium is detailed in Materials and Methods. StemFit: StemFit AK03N; P/S: penicillin/streptomycin; B27: B27 supplement; B27 INS (-): B27 supplement without insulin; Y: Y-27632; CHIR: CHIR99021; PF68: Pluronic F68; VC PMS: ascorbic acid phosphate magnesium salt; KGF: keratinocyte growth factor; LDN: LDN-193189; EGF: epidermal growth factor; NAM: nicotinamide; K-CYC: 3-keto-N-(aminoethyl-aminocaproyl-dihydrocinnamoyl)cyclopamine; PDBu: Phorbol 12,13-dibutyrate; ALK5i II: activin receptor-like kinase 5 inhibitor II; T3: triiodothyronine; XAV: XAV939; PD: PD-166866; BSA: bovine serum albumin; ITS-X: insulin, transferrin, selenium, ethanolamine solution; TR: TR06141363; NAC: N-acetyl cysteine.

Supplementary Table S2. List of primary antibodies used for flow cytometry analysis.

Antigen	Species	Source	Dilution
SOX17	Goat	AF1924; R&D systems, Minneapolis, MN	1:100
SOX2	Rabbit	3579S; Cell Signaling Technology, Danvers, MA	1:500
PDX1	Goat	AF2419; R&D systems, Minneapolis, MN	1:200
NKX6.1	Rabbit	54551S; Cell Signaling Technology, Danvers, MA	1:600
NKX6.1	Mouse	F55A12; DSHB, Iowa City, IA	1:417
C-peptide	Rat	GN-ID4; DSHB, Iowa City, IA	1:600
CHGA	Rabbit	ab68271; Abcam, Cambridge, UK	1:500
Ki67	Mouse	556003; BD Biosciences, San Jose, CA	1:100

Supplementary Video S1. Releasing the air bubbles trapped in each microwell. A small-scale bag having well diameters of φ 0.50 mm was filled with culture medium and incubated for 1 day with pressure applied. The video corresponds to Supplementary Fig. 2A. Scale bar = 200 μm.

Supplementary Video S2. Time-lapse images of cluster formation of induced pluripotent stem cell-derived pancreatic islet cells (iPICs) in a microwell bag. iPICs were seeded in a small-scale microwell bag with well diameters of 0.50 mm and cultured for 4 days. Scale bar = 200 μm.

Supplementary Video S3. Time-lapse of cluster formation of induced pluripotent stem cell-derived pancreatic islet cells (iPICs) in a large-scale bag. iPICs were seeded in a large-scale microwell bag with well diameter of 0.35 mm and cultured for 4 days. Scale bar = 200 μm.

Supplementary Video S4. Harvesting induced pluripotent stem cell-derived pancreatic islet cell (iPIC) clusters from a large-scale microwell bag. After culturing, the large-scale bag was detached from the jig and placed upside down to float clusters from inside of each microwell. Next, after injecting 400 mL air into the bag using a syringe, a drain tube was connected to the port. Thereafter, the bag was hung on a stand, and the culture solution was passed through a cell strainer and collected in two centrifuge tubes.
